# A Method of Transoral Finger Dissection for a Giant Epiglottic Lipoma

**DOI:** 10.1155/2014/640704

**Published:** 2014-10-27

**Authors:** Toshizo Koizumi, Katsunari Yane, Toshiaki Yamanaka, Tadashi Kitahara

**Affiliations:** ^1^Department of Otorhinolaryngology, Saiseikai Gose Hospital, 20 Mimuro, Gose, Nara 639–2306, Japan; ^2^Department of Otorhinolaryngology, Kinki University Nara Hospital, Ikoma, Nara, Japan; ^3^Department of Otorhinolaryngology and Head & Neck Surgery, Nara Medical University, Kashihara, Nara, Japan

## Abstract

*Background.* Subcutaneous lipomas that occur in the trunk and proximal extremities are commonly dissected by low-invasive method. However, a standard surgical method for lipomas of the epiglottis has been absent. Microscopic laryngeal surgery is appropriate to extirpate small epiglottic lipomas. However, microscopic laryngeal surgery may be insufficient for giant epiglottic lipomas because there is restricted visualization of the operating field of the tumor under the microscope. Furthermore, microscopic surgical instruments are very small to manipulate giant lipomas, and it would be excessive to approach these lipomas via external cervical incisions. *Case Presentation.* A 57-year-old female presented with a giant lipoma on the lingual surface of the epiglottis. Following a tracheotomy, microscopic surgery was inadequate to manipulate the epiglottic lipoma. Instead, we performed macroscopic surgery in which the epiglottic lipoma was pulled into the oral cavity with forceps and then separated from the surrounding tissues using the surgeon's finger to dissect the tumor en bloc. *Conclusion.* The low-invasive method of transoral finger dissection enabled the giant lipoma to be extirpated without leaving any remnants or causing excessive epiglottic damage.

## 1. Introduction

Surgical methods for epiglottic tumors are dependent on whether they are cystic nodules or noncystic solid masses [1–3]. Conventionally, an epiglottic cyst is dissected en bloc, or its wall is partially fenestrated if dissection is difficult. However, a standard surgical method for a solid epiglottic tumor is absent. The instruments for microscopic laryngeal surgery are appropriate to manipulate small epiglottic tumors [2, 4]. However, if the tumor is gigantic, the instruments may be too small to manipulate it, and its size restricts visualization of the operating field under the microscope. Instead of microscopic surgery, macroscopic surgery has been performed to approach and manipulate giant epiglottic tumors via high-invasive methods with external cervical incisions [1, 3, 5]. This level of invasiveness may be excessive for benign tumors such as lipomas. In this paper, we report on a low-invasive method of a transoral macroscopic surgery for a case of a giant epiglottic lipoma.

## 2. Case Report 

**Figure 1:**
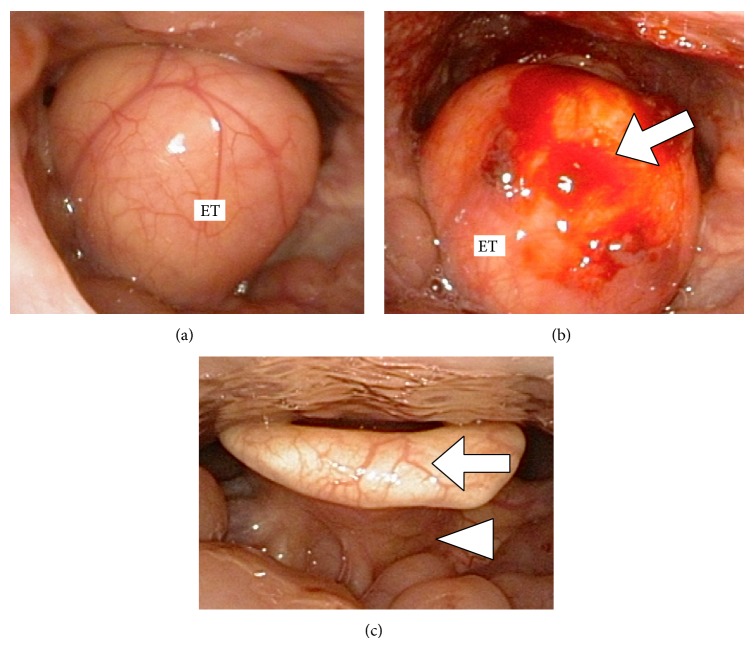
Findings of flexible laryngeal endoscopy. (a) The root of the tumor was unable to be visually distinguished to arise from the lingual or laryngeal surface. (b) No liquid was drained from the tumor after the anterior wall was punctured (arrow). (c) The epiglottis was not deformed (arrow), and its lingual surface was free from postoperative recurrence of the lipoma (arrow head).

**Figure 2:**
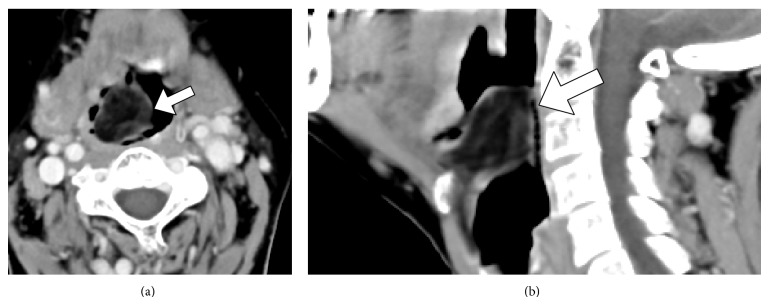
Contrast-enhanced (a) axial and (b) sagittal CT images showed a lipoma occurring on the lingual surface of the epiglottis (arrows).

A 57-year-old woman presented with a 5-year history of a progressive foreign-body sensation in the throat and a change in her voice. She reported no dysphagia and/or dyspnea and she was a nonsmoker. Flexible laryngeal endoscopy (Figure 1(a)) revealed a single, round tumor covered with smooth mucosa in the supraglottis. Although the tumor precluded visualization of the glottic gap, deliberate endoscopic manipulations revealed that the vocal cords were intact. To diagnose the epiglottic tumor in the simplest manner, we used forceps to partially puncture its anterior wall under local anesthesia (Figure 1(b)) and noted that liquid contents did not drain from it. Therefore, it was diagnosed as solid rather than cystic. Subsequently, contrast-enhanced computed tomography (CT) scans (Figure 2) showed an approximately 4 cm diameter tumor with a wide base on the lingual surface of the epiglottis. The internal density of the tumor was consistent with adipose tissue and the CT contrast effects were only observed in the peritumor regions. Consequently, the tumor was suspected to be a lipoma that had developed on the lingual surface of the epiglottis. Considering its potential to obstruct the supraglottic airway, the giant tumor was immediately treated by surgical intervention.

**Figure 3:**
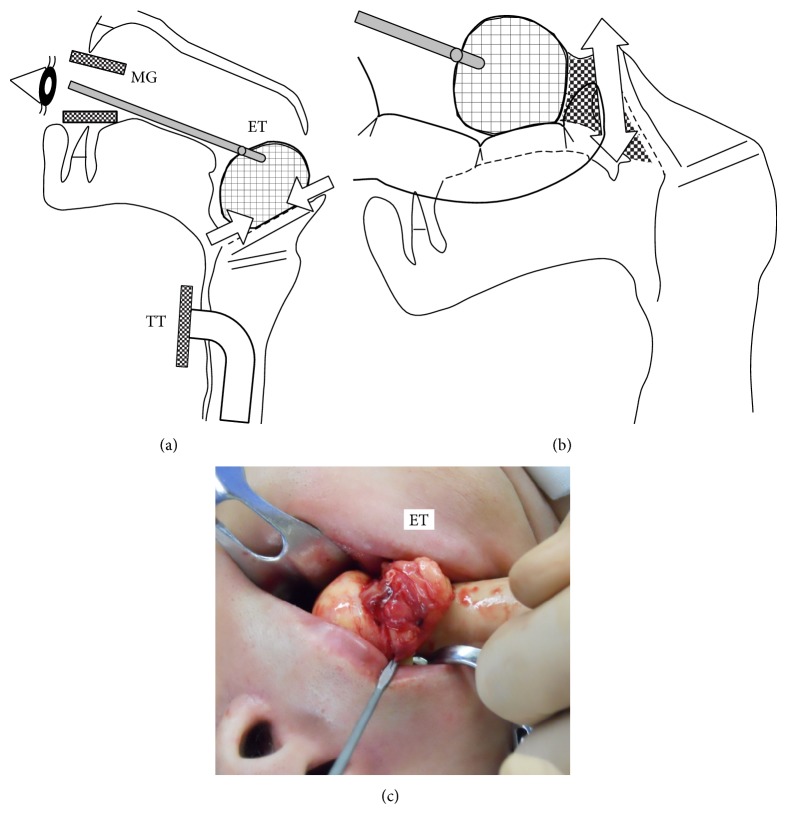
Illustrations and a photograph of transoral finger dissection. (a) After a tracheotomy and cessation of microscopic laryngeal surgery, the patient's mouth was opened with a large mouth gag (MG). Under macroscopic observation while maintaining the airway with a tracheostomy tube (TT), the epiglottic tumor (ET) was pulled into the oral cavity by grasping its frontal wall with forceps. The root of the lipoma attached to the epiglottis was slightly incised using a scalpel (arrows). (b) Traction was placed on the lipoma while simultaneously elevating the epiglottis and larynx to generate a spatial gap between ET and epiglottic cartilage. ET was blindly separated from epiglottic cartilage using the surgeon's index finger inserted into the gap. Because mucosal bundles covering ET converge to the lateral sides of the epiglottic attachment, they were cut using an electric knife (double-headed arrow). Consequently, ET was dissected en bloc. (c) ET was pulled out of the oral cavity and was manipulated using the surgeon's index finger.

We performed a tracheotomy on the conscious patient prior to inducing general anesthesia. Subsequently, we proceeded with microscopic laryngeal surgery using a cylindrical direct laryngoscope. However, the operating field was poorly visualized because the tumor was too large. Moreover, the instruments for microscopic laryngeal surgery were too small to manipulate the giant tumor. Because of the difficulty in completing the microscopic surgery, we proceeded with macroscopic surgery (Figure 3). Following extirpation of the tumor, we inserted a direct laryngoscope to microscopically confirm that the surgical wounds were localized on the lingual surface of epiglottis, the epiglottic cartilage was not deformed, and the mucosal cutoff margins of the tumor were scarcely bleeding. Finally, we sharply trimmed the mucosal remnants of the tumor to cover the epiglottic wounds without necessitating sutures or fibrin glue. We maintained the tracheostomy tube after the surgery was completed and it was removed 3 days postoperatively because there was no swelling associated with the epiglottic surgical wounds. The patient was discharged 10 days postoperatively.

The surgical specimen was a clearly marginated yellowish tumor measuring 40 × 39 × 36 mm. Histopathologically, the tumor was composed of mature adipocytes covered with multiple fibrous capsules with no evidence of malignancy. These findings established a diagnosis of epiglottic lipoma on the lingual surface.

The 1-year postoperative course has been uneventful, and, subjectively, the symptoms associated with the throat have disappeared. There has been no recurrence of the epiglottic lipoma (Figure 1(c)).

## 3. Discussion

Lipomas are benign tumors found in tissues with several subepithelial adipocytes [3, 6]. Lipomas often occur in the head and neck regions but also occasionally in the larynx, including the epiglottis and aryepiglottic fold [5, 7]. An epiglottic lipoma may originate from adipose tissues in the preepiglottic space, which is situated under the lingual surface of the epiglottis [5, 7, 8]. Similar to that in the current case, epiglottic lipomas almost always occur on the lingual surface (Figures 1(a) and 2) and rarely on the laryngeal surface of the epiglottis where adipocytes are absent. A simple method to diagnose whether an epiglottic tumor is cystic or solid is to puncture its anterior wall using small forceps (Figure 1(b)). Because the liquid contents did not drain from the epiglottic tumor in the current case, it was suspected to be solid. Furthermore, CT or magnetic resonance images can be useful to differentiate a solid tumor from a lipoma, pleomorphic adenoma, neuroma, hemangioma, chondroma, fibroma, or malignant neoplasm of liposarcoma [9, 10]. The current case was preoperatively suspected to be a lipoma because of its CT images (Figure 2). Although small epiglottic lipomas are asymptomatic, large lipomas cause symptoms such as a foreign-body sensation of the throat, dysphagia, and dysphonia due to an obstruction of the supraglottic airway [2–5]. Moreover, the epiglottic lipoma in the current case was large enough to disturb endoscopic visualization of the glottis. A giant epiglottic lipoma should be immediately treated because of the risk of sudden life-threatening blockage of the airway.

Lipomas that occur in the trunk and proximal extremities are commonly extirpated by low-invasive methods [9, 10]. The conventionally performed method for subcutaneous lipomas is to use the surgeon's index finger to separate it from surrounding tissues and dissect it en bloc [10]. However, a standard surgical method for epiglottic lipomas remains absent. Microscopic laryngeal surgery using small instruments is appropriate to manipulate small epiglottis lipomas. However, the instruments for microscopic laryngeal surgery are too small to manipulate giant epiglottic lipomas. In previous reports, giant epiglottic lipomas have been dissected by macroscopic surgery via external cervical incisions (e.g., thyrotomy, transhyoid or lateral pharyngotomy, or laryngofissure) [1, 3, 5] or piecemeal excisions [9, 11]. A piecemeal excision is inappropriate because remnants or dissemination of the lipoma can cause postoperative recurrence [9, 11, 12]. In the current case, the method of transoral finger dissection satisfied the conditions that the giant epiglottic lipoma was dissected en bloc in a low-invasive manner. In addition, surgical manipulations with the surgeon's finger allowed tactual evaluation of the entire structure of the lipoma, particularly deep within the surgical field, and avoided excessive damage to neurovascular structures and the epiglottic cartilage [9, 10]. Although it was invasive, the tracheotomy was indispensable in the current case because of the risk of blockage of the supraglottic airway both intra- and postoperatively [4]. Furthermore, the tracheotomy provided an increased area for surgical manipulations because it allowed the patient's oral cavity to be free of a tracheal tube.

Little is known about how well the epiglottic cartilage and larynx tolerate the pressure of being pulled on. In general, the distance of laryngeal elevation that accompanies swallowing in normal adults is approximately 35 mm [13]. Prolonged epiglottic and laryngeal elevation may be advantageous to manipulate the epiglottic lipoma into larger working space of the oral cavity. In the current case, general anesthesia possibly both contributed to relaxation of the muscles that support the laryngeal structures and simplified the elevation of the epiglottis and larynx including the lipoma. In addition, the epiglottis is constructed of an elastic cartilage, which has both deformability and sufficient tensile strength [14]. In the current case, the lengthy periods of epiglottic stretching did not result in postoperative deformations or damage to the epiglottis.

## 4. Conclusion

A low-invasive surgical method was used to treat a giant epiglottic lipoma in a 57-year-old female. Microscopic laryngeal surgery after a tracheotomy was inadequate to manipulate the lipoma. Instead, macroscopic surgery was performed in which we used forceps to pull the lipoma into the oral cavity and then we separated it from the surrounding tissues using the surgeon's index finger. This method of transoral finger dissection was advantageous to dissect the giant lipoma en bloc without excessive damage to the epiglottis.
